# Durable disease control in a radiation-induced high-grade glioma harboring *NF1* and *PTPN11* co-mutations

**DOI:** 10.1093/noajnl/vdag053

**Published:** 2026-02-27

**Authors:** Mohamed Sherief, Maria Fatteh, Jaime Wehr, Matthias Holdhoff, Charles G Eberhart, Valsamo Anagnostou, Karisa C Schreck

**Affiliations:** Department of Oncology, Sidney Kimmel Comprehensive Cancer Center, Johns Hopkins University School of Medicine, Baltimore; The Johns Hopkins Molecular Tumor Board, Johns Hopkins University School of Medicine, Baltimore; Department of Oncology, Sidney Kimmel Comprehensive Cancer Center, Johns Hopkins University School of Medicine, Baltimore; The Johns Hopkins Molecular Tumor Board, Johns Hopkins University School of Medicine, Baltimore; Department of Oncology, Sidney Kimmel Comprehensive Cancer Center, Johns Hopkins University School of Medicine, Baltimore; The Johns Hopkins Molecular Tumor Board, Johns Hopkins University School of Medicine, Baltimore; Department of Oncology, Sidney Kimmel Comprehensive Cancer Center, Johns Hopkins University School of Medicine, Baltimore; Department of Pathology, Johns Hopkins University School of Medicine, Baltimore; Department of Oncology, Sidney Kimmel Comprehensive Cancer Center, Johns Hopkins University School of Medicine, Baltimore; The Johns Hopkins Molecular Tumor Board, Johns Hopkins University School of Medicine, Baltimore; Department of Oncology, Sidney Kimmel Comprehensive Cancer Center, Johns Hopkins University School of Medicine, Baltimore; Department of Neurology, Johns Hopkins University School of Medicine, Baltimore

**Keywords:** MAPK targeted therapy, MEK inhibitor, NF1, radiation induced glioma, SHP2

## Abstract

Radiation-induced gliomas (RIGs) are rare and aggressive secondary brain tumors arising years after cranial irradiation. Their management remains challenging due to prior radiation exposure, which limits additional radiation, and a lack of effective chemotherapies. Recent studies have revealed distinct molecular profiles in RIGs with unclear clinical implications. This study presents the case of an individual who developed a high-grade glioma three decades after curative craniospinal radiation for medulloblastoma. He was treated with repeat radiation and temozolomide chemotherapy but developed recurrence with disseminated leptomeningeal disease thereafter. Molecular profiling of the tumor revealed a loss-of-function *NF1* mutation and a gain-of-function *PTPN11* mutation, two convergent alterations in the MAPK pathway. Based on these findings, the patient was treated with a MEK inhibitor, trametinib, and achieved durable disease control for 20 months until progression. This case underscores the importance of genomic profiling in RIGs and potential utility of molecularly targeted approaches in this population.

## Introduction

Radiation-induced gliomas (RIGs) are a rare yet serious late complication of cranial irradiation, often arising years or decades after the initial exposure. These tumors typically present with high-grade histopathology (eg glioblastoma [GBM]) and carry a dismal prognosis, with a reported median overall survival averaging 7 to 11 months.^1^ In the absence of specific guidelines for RIGs, current management strategies largely reflect those of *de novo* high-grade glioma (HGG), typically involving re-irradiation and chemotherapy. Upon the inevitable disease progression, options become significantly limited, as additional radiation is often unfeasible due to prior exposure and surgical re-resection is challenging. Salvage chemotherapy provides only marginal benefits, highlighting the need for novel therapeutic strategies in these patients, particularly those guided by tumor molecular profiling.

In recent years, comprehensive genomic characterization of RIGs has indicated that RIGs harbor distinct genetic alterations compared to their *de novo* counterparts.^2–4^ These include recurrent structural alterations such as *PDGFRA* and *CDK4* amplifications and *CDKN2A/B* homozygous deletions, as well as mutations in the mitogen-activated protein kinase (MAPK) pathway genes including *NF1*.^2–4^ Classical alterations associated with spontaneous HGGs such as *IDH1/2*, *PTEN*, or *TERT* are infrequent in RIGs. Despite advances in comprehensive genomic profiling, due to the rarity of this tumor, no clinical trials have specifically evaluated targeted therapies for RIGs, and most patients continue to rely on conventional treatments that yield suboptimal outcomes.

Here, we present the case of a patient with a high-grade RIG harboring a loss-of-function (LOF) mutation in *NF1* and a gain-of-function (GOF) mutation in *PTPN11*, both key regulators of the MAPK pathway. Given this molecular profile, the patient was treated with a MEK inhibitor, trametinib, at time of disease recurrence, attaining disease control for 20 months. This case highlights the importance of genomic profiling in RIGs and the potential utility of molecularly targeted approaches for patients with RIGs.

## Case Report

The patient is a 45-year-old male with a history of medulloblastoma, of unknown molecular subtype, diagnosed at age 10. He underwent gross total resection, followed by craniospinal irradiation (CSI; 3600 cGy with a posterior fossa boost to 5600 cGy), and adjuvant chemotherapy with vincristine, lomustine, and cisplatin. At age 20, he was diagnosed with a right frontal meningioma, presumed to be radiation-induced, for which he underwent curative surgical resection.

**Figure 1.**
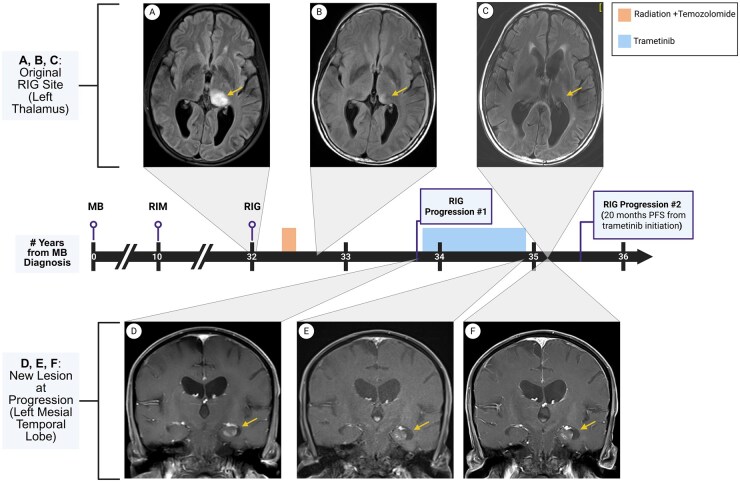
Timeline of diagnosis, treatment, and radiographic response to trametinib. (A) T1-weighted post-gadolinium MRI of the original lesion in the left thalamus at initial presentation, (B) 3 months post-chemoradiation showing treatment response, and (C) 15 months after the initiation of trametinib showing stability. (D) A new lesion in the left mesial temporal lobe was identified 15 months post-chemoradiation, showing disease progression (Progression #1). Treatment with trametinib was initiated, and MRI (E) after 12 months of treatment and (F) 3 months after trametinib discontinuation confirmed stable disease, prior to a second progression that occurred 20 months after trametinib initiation (Progression #2). Abbreviations: MB, medulloblastoma; RIM, radiation-induced meningioma; RIG, radiation-induced glioma; PFS, progression-free survival; MRI, magnetic resonance imaging. Created in BioRender. Schreck, K. (2026) https://BioRender.com/57ob9ut.

At age 42, 32 years after his initial medulloblastoma diagnosis, the patient developed progressive leftward gaze dysfunction, headaches, and worsening left-sided motor deficits. Brain MRI revealed a left thalamic FLAIR -hyperintense lesion that increased in size over 2 months, prompting further evaluation with a stereotactic biopsy (Figure 1A). Histopathologic evaluation showed features consistent with high-grade astrocytic glioma, IDH-wildtype. To further classify the tumor, molecular profiling was performed. FISH analysis revealed a 1p deletion with intact 19q. Next‑generation sequencing (NGS) using the Caris MI Profile comprehensive assay, which incorporates whole-exome and whole-transcriptome sequencing, detected a pathogenic *NF1* p.R681* (variant allele frequency [VAF], 62%) and *PTPN11* p.T507K (VAF, 32%) mutation, along with an unmethylated MGMT promoter. The tumor mutation burden was low (5 mutations/Mb) and PD-L1 immunohistochemistry was negative. Given the patient’s history of CSI, the histopathology and molecular profiling supported an integrated histopathologic diagnosis of high-grade RIG, consistent with previous studies reporting similar molecular alterations in RIGs.^2^^,^^3^ The tumor was classified as high-grade with a Ki-67 proliferation index exceeding 20%.

The patient completed a 6-week course of proton radiotherapy to a total dose of 5460 cGyE administered concurrently with temozolomide. Proton therapy was selected given the patient’s prior cranial irradiation, with the goal of minimizing cumulative radiation exposure to surrounding normal brain tissue while adhering to dose constraints for adjacent critical structures, including the brainstem. Given the tumor’s unmethylated MGMT promoter status that typically confers resistance to temozolomide, adjuvant temozolomide was deferred, and he transitioned to active surveillance. Initial follow-up MRI demonstrated early tumor response with continued mass shrinkage (Figure 1B). However, at 15 months post-chemoradiation, surveillance MRI revealed disease progression with the emergence of two new lesions: an 8 mm enhancing nodule in the left mesial temporal lobe and a 3-5 mm enhancing nodule along the ependymal surface of the left occipital horn, with no progression at the primary site (Figure 1C and D). These were concerning for leptomeningeal dissemination given their proximity to the lateral ventricle.

Given the presence of *NF1* and *PTPN11* mutations, both of which drive constitutive MAPK activation and were deemed oncogenic by the Johns Hopkins Molecular Tumor Board, targeted therapy was recommended. Additionally, given the occurrence of two radiation-associated neoplasms, comprehensive germline testing was recommended; however, the patient and family declined. The patient did not meet eligibility criteria for a clinical trial and therefore began off-label monotherapy with the MEK inhibitor trametinib at the FDA-approved dose of 2 mg daily. After two months of treatment, he developed drug-induced pneumonitis, prompting a temporary treatment hold for 1 month. Following resolution of treatment-related toxicity, he resumed trametinib at a reduced dose of 1 mg daily and continued therapy for a total of 12 months before discontinuation due to declining functional status and the duration of treatment. MRI at the time of discontinuation demonstrated stable disease in the left mesial temporal lobe lesion and complete disappearance of the left occipital horn ependymal nodule, with no evidence of new enhancement or progression (Figure 1E). Follow-up MRI 3 months post-discontinuation confirmed continued stable disease (Figure 1F). Twenty months after initiating trametinib and seven months after drug discontinuation, the patient developed two new ependymal nodules consistent with disease progression.

## Discussion

### MAPK Pathway Activation in Gliomas is Clinically Actionable

The MAPK pathway plays a central role in gliomagenesis and progression and can be activated through upstream RTK alterations, loss of negative regulators such as NF1, or activating mutations in effectors like *BRAF*. Given frequent dysregulation, the MAPK pathway has emerged as a therapeutic target in gliomas. MEK inhibitors such as trametinib and selumetinib have demonstrated notable clinical benefit in a select low-grade glioma (LGG) subtypes, particularly pediatric gliomas with *BRAF* V600E mutations or arising in the context of an NF1 predisposition syndrome.^5^^,^^6^ There are no published clinical data on the use of MEK inhibitors in RIGs, a tumor type that, based on recent molecular profiling studies, appears molecularly distinct from de novo HGGs, with frequent MAPK pathway alterations, and follows an aggressive clinical course.^2–4^ Here, we report the case of a patient with a high-grade RIG harboring concurrent *NF1* and *PTPN11* mutations who attained durable disease control following treatment with trametinib.

### NF1 and PTPN11 Co-Mutation May Confer MAPK Pathway Dependence and Sensitivity to MEK Inhibition in HGGs

**Figure 2.**
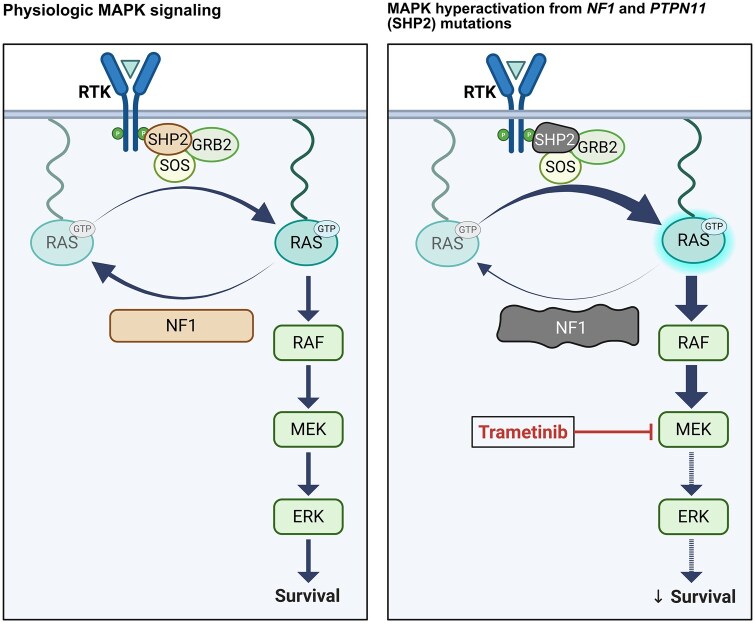
Schematic of MAPK pathway activation and therapeutic inhibition in *NF1*/*PTPN11*-mutated glioma. Under physiological conditions, MAPK signaling is tightly regulated. NF1 negatively regulates RAS activity, and SHP2 (encoded by *PTPN11*) remains autoinhibited until activated by upstream RTKs (left panel). In tumors with *NF1* loss and activating *PTPN11* mutations, RAS becomes constitutively active, leading to sustained MAPK signaling; MEK inhibition by trametinib reduces ERK activation and impairs downstream survival signaling (right panel). Abbreviations: RTK, receptor tyrosine kinase; MAPK, mitogen-activated protein kinase; NF1, neurofibromin 1; PTPN11, protein tyrosine phosphatase non-receptor type 11; SHP2, Src homology region 2-containing protein tyrosine phosphatase-2. Created in BioRender. Schreck, K. (2026) https://BioRender.com/57ob9ut.

The rationale for using MEK inhibition in this case stems from the biological consequences of the *NF1* p.R681* truncating mutation identified in the tumor. *NF1* encodes the RAS-GTPase activating protein neurofibromin, a negative regulator of RAS, and its loss leads to constitutive RAS activation and sustained MAPK signaling (Figure 2). *NF1* mutations are well-documented in both RIGs and de novo HGGs, where they cluster with the mesenchymal subtype of GBM.^2^ Preclinical studies in *NF1*-deficient GBM models have demonstrated sensitivity to MEK inhibition through suppression of MAPK activity and reduced tumor proliferation.^7^^,^^8^ Clinically, however, only a few reports have documented modest responses to trametinib monotherapy in *NF1*- mutated HGGs, particularly GBM. For instance, a subprotocol of the NCI-MATCH trial reported that among five GBM patients with deleterious *NF1* alterations, two showed clinical benefit, with the longest reported PFS reaching 9.2 months.^9^

While *NF1* LOF provides a compelling rationale for targeting MAPK signaling, the presence of a concurrent activating *PTPN11* mutation further reinforces MAPK pathway dependence and introduces additional considerations for pathway regulation and therapeutic responsiveness. A recent multi‑omic study identified *PTPN11* and its protein phosphorylation status as a central node of oncogenic signaling in HGGs, and clinical evidence further suggests that mutations in *PTPN11* are associated with poor prognosis in GBM.^10^  *PTPN11* encodes SHP2, a protein tyrosine phosphatase that normally remains in an autoinhibited conformation. Upon stimulation by phosphorylated RTKs, SHP2 transitions to an active conformation, regulating multiple downstream signaling pathways, including the MAPK pathway (Figure 2).^11^ GOF mutations, such as the *PTPN11* p.T507K that lies in the phosphatase domain, can disrupt SHP2’s autoinhibition, and lead to constitutive phosphatase activity, resulting in sustained RAS pathway signaling, and additional input into the MAPK cascade, amplified by NF1 loss.

While this convergence on MAPK signaling may enhance sensitivity to MEK inhibition, it also raises questions regarding potential mechanisms of resistance. Preclinical studies, including those in *NF1*-deficient cancers and *BRAF* V600E-mutant HGGs, have shown that MEK inhibitor resistance can emerge through loss of negative feedback leading to activation of upstream RTKs, a process in which SHP2 upregulation plays a central role.^12^^,^^13^ These observations prompted ongoing preclinical and clinical investigations into dual MEK and SHP2 inhibition as a strategy to overcome adaptive resistance to MAPK pathway inhibition, though no SHP2 inhibitors are yet FDA approved. It remains unknown whether combined therapy would prevent adaptive resistance in cancers with combined *NF1* and *PTPN11* alterations.

### NF1 and PTPN11 Co-Mutations are Enriched in RIGs but also Present in a Subset of de novo HGGs

Although RIG is a rare subset of HGG, a similar *NF1*/*PTPN11* co-mutation pattern has been previously reported in some of the few available genomic studies of this entity. For example, Whitehouse et al. described a patient with a RIG harboring both *NF1* and *PTPN11* mutations, in which the corresponding patient-derived xenograft exhibited high levels of phosphorylated ERK, consistent with MAPK pathway activation.^14^ This observation was further supported by one of the largest genomic analyses of RIG to date, in which DeSisto et al. identified *NF1* mutations in 9 of 32 patients (28.1%), *PTPN11* mutations in 4 (12.5%), and *NF1*/*PTPN11* co-mutations in 3 patients (9.3%).^2^ Notably, in the same study, trametinib emerged as one of the most active agents in an *in vitro* drug screen of RIG-derived cell lines, although drug responses were not stratified by mutational profile.

**Figure 3.**
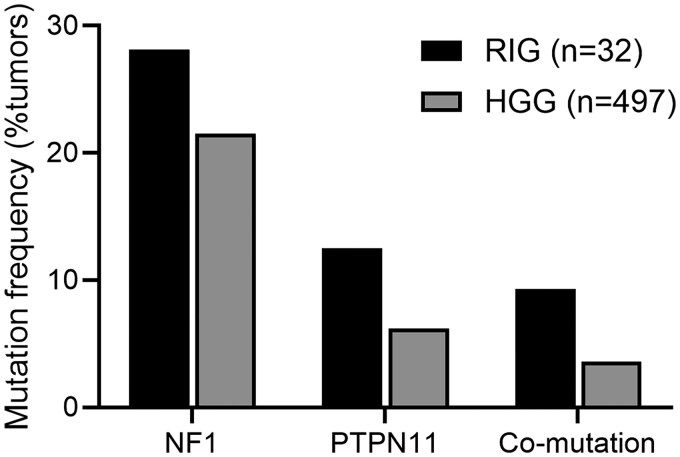
Frequency of *NF1* and *PTPN11* mutations in RIG and sporadic HGG. Percentage of tumors with *NF1* (*n*=9 out of 32, 28.1%), *PTPN11* (*n*=4 out of 32, 12.5%), or *NF1* + *PTPN11* co-mutations (*n*=3 out of 32, 9.3%) in RIGs (*n*=32) are shown alongside frequencies of tumors with *NF1* (*n*=107 out of 497, 21.5%), *PTPN11* (*n*=31 out of 497, 6.2%), or *NF1* + *PTPN11* co-mutations (*n*=18 out of 497, 3.6%) in sporadic HGG (*n*=497). RIG data were derived from the DeSisto et al cohort; HGG data were obtained from the MSKCC glioma dataset via cBioPortal. Abbreviations: RIG, radiation-induced glioma; HGG, high-grade glioma.

Whether this therapeutic vulnerability to MEK inhibition, observed in this case of *NF1/PTPN11* co-mutated RIG, represents a molecular synergy unique to RIGs or extends to a broader subset of de novo HGGs, a more common and molecularly heterogeneous tumor type, remains to be determined. To further explore the occurrence of *NF1*/*PTPN11* co-mutations, we queried a publicly available dataset of GBMs from the MSKCC glioma cohort via cBioPortal (http://www.cbioportal.org). Among 497 GBM tumors, *NF1* mutations were identified in 107 (21.5%), *PTPN11* mutations in 31 (6.2%), and co-mutations in 18 tumors (3.6%). While the co-mutation frequency was lower than that observed in DeSisto RIG cohort (9.3%), its presence suggests that this alteration pattern is not unique to RIGs (Figure 3). Notably, this co-occurrence pattern aligns with findings from a recent analysis of *NF1*-mutant GBMs, which reported that tumors with *NF1* mutations were significantly more likely to harbor co-mutations in *PTPN11* compared to *NF1* wild-type tumors. Together, these findings raise the possibility that a subset of *de novo* HGGs may share similar MAPK pathway dependencies—and potentially similar sensitivity to MEK inhibition—as observed in this case.

### Potential for MEK Inhibition as a Radiosensitization Strategy in NF1/PTPN11-Mutated RIG

Building on recent preclinical work by Ioannou et al., which demonstrated that MEK inhibition enhances radiotherapy efficacy in *NF1*-deficient GBM models, one of which also harbors a *PTPN11* alteration, and earlier studies showing that SHP2 inhibition increases glioma radiosensitivity, our observation of trametinib’s benefit in an *NF1/PTPN11*-mutated RIG suggests this synergy could extend to RIGs.^15^ Further work will be necessary to understand whether combining MEK inhibitors with radiotherapy could enhance tumor control or allow for radiation dose de-escalation, given the prior radiation exposure in RIGs patients.

This case highlights the value of comprehensive molecular profiling in RIGs and presents clinical evidence for MEK inhibitor activity in a MAPK pathway-addicted RIG. While single-patient outcomes must be interpreted with caution, the observed 20-month disease control period in this case supports the therapeutic potential of targeting MAPK signaling in *NF1/PTPN11* co-mutated gliomas. Future studies are needed to validate these findings and explore rational treatment strategies, such as dual MEK/SHP2 inhibition and radiosensitization approaches, in both *de novo* and secondary HGGs harboring similar molecular features.

## Data Availability

The datasets analyzed in the current study are available in the cBioPortal repository for Cancer Genomics (https://www.cbioportal.org/).

## References

[1] Onishi S , Yamasaki F, Amatya VJ, et al Characteristics and therapeutic strategies of radiation-induced glioma: case series and comprehensive literature review. J Neurooncol. 2022;159:531-538. 10.1007/s11060-022-04090-9 35922583

[2] DeSisto J , Lucas JT Jr., Xu K, et al Comprehensive molecular characterization of pediatric radiation-induced high-grade glioma. Nat Commun. 2021;12:5531. 10.1038/s41467-021-25709-x 34545084 PMC8452624

[3] Deng MY , Sturm D, Pfaff E, et al Radiation-induced gliomas represent H3-/IDH-wild type pediatric gliomas with recurrent PDGFRA amplification and loss of CDKN2A/B. Nat Commun. 2021;12:5530. 10.1038/s41467-021-25708-y 34545083 PMC8452680

[4] López GY , Van Ziffle J, Onodera C, et al The genetic landscape of gliomas arising after therapeutic radiation. Acta Neuropathol. 2019;137:139-150. 10.1007/s00401-018-1906-z 30196423 PMC6589431

[5] Bouffet E , Geoerger B, Moertel C, et al Efficacy and Safety of Trametinib Monotherapy or in Combination With Dabrafenib in Pediatric BRAF V600-Mutant Low-Grade Glioma. *J Clin Oncol*. 2023;41: 664-674. 10.1200/JCO.22.01000 36375115 PMC9870224

[6] Fangusaro J , Onar-Thomas A, Young Poussaint T, et al Selumetinib in paediatric patients with BRAF-aberrant or neurofibromatosis type 1-associated recurrent, refractory, or progressive low-grade glioma: a multicentre, phase 2 trial. LancetOncology. 2019;20:1011-1022. 10.1016/S1470-2045(19)30277-3 PMC6628202 31151904

[7] See WL , Tan IL, Mukherjee J, Nicolaides T, Pieper RO. Sensitivity of glioblastomas to clinically available MEK inhibitors is defined by neurofibromin 1 deficiency. Cancer Res. 2012;72:3350-9. 10.1158/0008-5472.CAN-12-0334 22573716 PMC4128256

[8] Schreck KC , Allen AN, Wang J, Pratilas CA. Combination MEK and mTOR inhibitor therapy is active in models of glioblastoma. Neurooncol Adv. 2020;2:vdaa138. 10.1093/noajnl/vdaa138 33235998 PMC7668446

[9] Wisinski KB , Flamand Y, Wilson MA, et al Trametinib in patients with NF1-, GNAQ-, or GNA11-mutant tumors: results from the NCI-MATCH ECOG-ACRIN trial (EAY131) Subprotocols S1 and S2. JCO Precis Oncol. 2023;7:e2200421. 10.1200/PO.22.00421 37053535 PMC10309549

[10] Liu J , Cao S, Imbach KJ, et al Multi-scale signaling and tumor evolution in high-grade gliomas. Cancer Cell. 2024;42:1217-1238 e19. 10.1016/j.ccell.2024.06.004 38981438 PMC11337243

[11] Sodir NM , Pathria G, Adamkewicz JI, et al SHP2: a pleiotropic target at the interface of cancer and its microenvironment. Cancer Discov. 2023;13:2339-2355. 10.1158/2159-8290.CD-23-0383 37682219 PMC10618746

[12] Wang J , Pollard K, Allen AN, et al Combined inhibition of SHP2 and MEK is effective in models of NF1-deficient malignant peripheral nerve sheath tumors. Cancer Res. 2020;80:5367-5379. 10.1158/0008-5472.CAN-20-1365 PMC7739379 33032988

[13] Ayanlaja AA , Chang M, Lalwani K, et al Combined inhibition of SHP2 overcomes adaptive resistance to type 1 BRAF inhibitors in BRAF V600E-driven high-grade glioma. Neurooncol Adv. 2025;7:vdaf170. 10.1093/noajnl/vdaf170 40900823 PMC12400027

[14] Whitehouse JP , Howlett M, Hii H, et al A novel orthotopic patient-derived xenograft model of radiation-induced glioma following medulloblastoma. Cancers (Basel). 2020;12:2937. 10.3390/cancers12102937 PMC7600047 33053751

[15] Ioannou M , Lalwani K, Ayanlaja AA, Chinnasamy V, Pratilas CA, Schreck KC. MEK inhibition enhances the antitumor effect of radiotherapy in NF1-deficient glioblastoma. Mol Cancer Ther. 2024;23:1261-1272. 10.1158/1535-7163.MCT-23-0510 38714355 PMC11374499

